# How physiology solves the gene‐centric impasse

**DOI:** 10.1113/EP093664

**Published:** 2026-07-01

**Authors:** Denis Noble, Reine Bourret

**Affiliations:** ^1^ Department of Physiology, Anatomy & Genetics University of Oxford Oxford UK; ^2^ Daegu‐Gyeongbuk Institute of Advanced Studies Daegu South Korea; ^3^ Independent researcher Paris France

**Keywords:** biological relativity, CellML repository, central dogma, ivabradine, multiscale causation, procoralan, systems biology

## Abstract

The criteria for distinguishing between association and causation in studies of genes and function are clarified. Genomic association scores alone cannot satisfy those criteria, which is why treatment of common diseases has not benefitted from the cures expected when the Human Genome Project was launched. Instead, causal understanding of function and disease requires detailed experimental data at the relevant levels of organization in the organism, from which quantitative physiological modelling then enables causal processes to be measured accurately. They can then be compared with association scores. An example shows how this process works in the heart and how that work led to the identification of a useful medication. Another major field of clinical importance is the nervous system, because nervous diseases were also expected to yield to genomics‐driven discoveries of genetic cures. That expectation also has not been fulfilled. It is now necessary to investigate causation at functional physiological levels of organization in order to develop strategies that can identify cures for multifactorial diseases.

## Abstract (highlights)

- **What is the topic of this review?**
  The gene‐centric impasse and its causes are identified.
- **What advances does it highlight?**
  The methods by which physiology can bypass this impasse are analysed and explored as they might be applied to the diseases of the cardiovascular and nervous systems.

## 1 INTRODUCTION

In a previous article in *Experimental Physiology* on the principles of systems biology, we identified the gene‐centric impasse (Noble & Bourret, 2026). We now explain how that impasse arose and how it can be circumvented by physiological identification of the causes and cures. This will be a closely argued article and readers may appreciate orientation on the arguments in the various sections and how they interact and support the overall conclusions.

We begin by showing that the statistics on ageing populations and health‐care budgets have shown those budgets to balloon beyond the resources even of developed countries. If we were expecting the Human Genome Project to solve the common diseases of old age, that project has failed, leaving health services to pick up the exorbitant costs. We then explain why that project failed in its stated aims. That requires analysis of the mathematical conclusions of medical screening statistics, provided by the work of Nicholas Wald and his collaborators for >30 years now. We then show how physiological causation of functions and their disease states requires quantitative measures of dynamic processes. To illustrate this process, we analyse the way in which the analysis of multiple processes governing the heart pacemaker led to a safe medication, ivabradine (procoralan). New computations of the pacemaker model developed in 1992 are used to illustrate how that medication works as a demand limiter, protecting the hearts of ischaemic patients from fatal arrhythmia. Similar work, now available in the CellML Repository, might be used to identify causation and cures in many other areas of physiological investigation. We finish the article with examples in the nervous system where study of association scores has also failed to identify causation or cures. Such diseases are sometimes more diseases of the mind than of the brain alone. Finally, we return to the key fact that underlies both our previous article and this one: the reasons why the central dogma of molecular biology is neither central nor a dogma.

## 2 HOW DID THE GENE‐CENTRIC IMPASSE ARISE?

The gene‐centric impasse in health care has two main causes. First, contrary to the expectations of the Human Genome Project, the cures for common fatal diseases have not been discovered. Instead, mass genome sequencing has generated basic scientific information, which has enabled applications in a range of other fields, including detective and legal work, where DNA sequences are useful to identify individuals and their relationships; the refinement of the taxonomic trees of life and their great value in evolutionary biology; and in generating the basic catalogues and databases of molecular biology.

On the basic catalogues and databases, we have also discovered that more needs to be known about biology beyond the genome (Ball, 2023; Noble, 2006; Noble & Noble, 2023). The discovery that only ∼5% of the sequences form DNA templates for proteins has revealed the vast extent of the genome enabling the formation of innumerable RNAs used to control the genome, forming much of the regulatory networks that enable physiological functions to be generated. Those networks consist of proteins, RNAs, metabolites, hormones, transmitters, other signalling molecules and the variety of cellular and subcellular structures (organelles) that contain and constrain these networks. That is where we should be looking for the causes of health and disease.

DNA has been over‐privileged in the quantification of inheritance, because it is easy to represent the information digitally. The four bases, C, G, A and T, could be replaced with the noughts and ones of binary code, with each base represented in a 2‐bit code: 00, 01, 10 and 11. No information would be lost. By comparison, the rest of the inherited egg and sperm cells requires representation as analog information. The packed membraneous structures could be represented at many different levels of resolution. A conservative estimate of the resolution needed shows that the information content is larger than the genome (Noble, 2017) at a molecular scale of mapping the egg cell. Amplify a nucleotide to the size of a golf ball and the cell membrane would be hundreds of miles away!

**FIGURE 1:**
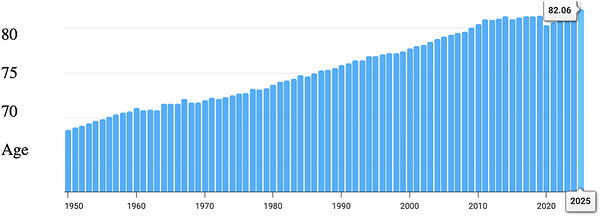
Life expectancy in the UK between 1950 and 2025. (From https://www.macrotrends.net/datasets/global‐metrics/countries/gbr/united‐kingdom/life‐expectancy.)

**FIGURE 2:**
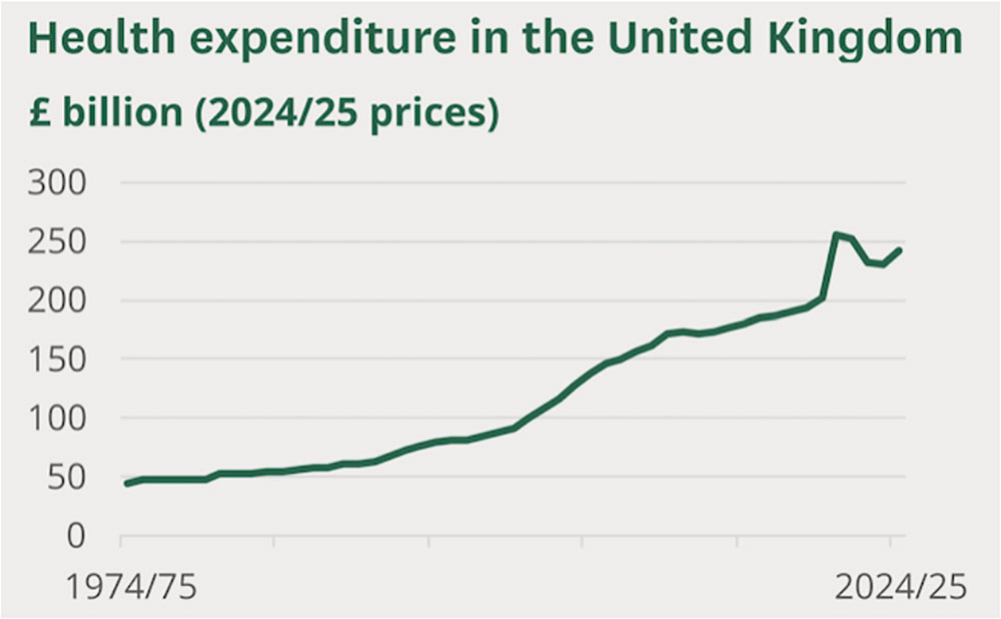
Health expenditure in the UK. Similar massive increases have occurred in many other developed countries (Bridges, 2026).

The second cause of the impasse is the great success of health care in developed countries. Instead of dying young, many people now reach old age and suffer the multifactorial diseases that have not succumbed to molecular‐level solutions (Figure 1; Table 1). The cost of the diseases of old age (cancer, cardiovascular diseases and nervous diseases, including the various forms of dementia) is stretching national health budgets way beyond what can be afforded. Genomics has been expensive, but the cost of failing to find cures for the diseases of old age is even more so (Figure 2). Clearly, it would have been convenient, to say the least, had genomics succeeded in curing these diseases.

**TABLE 1:** Life expectancy in the UK, Canada, France and Japan.

Year	UK	Canada	France	Japan
1950	68.69	68.29	66.00	60.64
1960	71.13	71.13	69.87	67.70
1970	71.97	72.70	71.66	71.92
1980	73.68	75.10	74.05	75.99
1990	75.88	77.44	76.60	78.84
2000	77.74	79.17	79.06	81.08
2010	80.40	81.32	81.66	82.84
2020	80.33	81.54	82.18	84.56
2025	82.06	83.26	83.39	85.27

## 3 WHY WERE THE CLINICAL PROMISES OF GENOMICS NOT FULFILLED?

One answer is that it was unrealistic even to expect it to happen. For that discovery, we have to thank Nicholas Wald and his collaborators, who have worked for decades to challenge the now popular use of polygenic scores and to demystify their application. Their work is important because when the Human Genome Project was launched, it was expected that the diseases of old age might involve only a few genes, leading to the prospect of genetic cures. It was also hoped that polygenic scores might enable screening to determine who would be at greatest risk of developing a disease of old age and guide possible preventative measures. Instead, we have found that hundreds of genes are associated with the debilitating illnesses of old age, and useful screening has not been forthcoming. It is precisely the expectation that it would that Wald's work has challenged.

Wald was the first editor of the *Journal of Medical Screening*, in 1994, and he is still the Editor‐in‐Chief. In editorials (e.g. Wald, 1994a, b; Wald & Dormandy, 1999) he has traced the problems facing the development of medical screening trials. In this section, we summarize the main findings that need to be understood.

In a 1999 paper, that is the same year as Francis Collins’ (1999) article on the great promises of the Human Genome Project, Wald et al. (1999) wrote in the *British Medical Journal* an answer to the question: ‘When can a risk factor be used as a worthwhile screening test?’. They pointed out:

The fact that a risk factor must be very strongly associated with a disorder if it is to be a worthwhile screening test is not widely recognized.

They explained:

The fact that a strong risk factor can be a poor screening test may seem counterintuitive. The paradox is explained when it is recognized that the relative odds (or relative risk), usually used to evaluate risk factors as possible causes of a disease, is usually assessed by comparing the risk of disease at each end of the distribution of the risk factor. … Even a relative odds of 200 between the highest and lowest fifths will yield a detection rate of no more than about 56% for a 5% false positive rate, provided, as is commonly the case, that the distribution of the screening variable is approximately Gaussian (or log Gaussian) and shows a similar SD in affected and unaffected people. (Wald et al., 1999)

We have therefore known that the strength of association found in genome‐wide association studies would need to be very high for the promises of the Human Genome Project to be fulfilled. Yet, instead, most association scores (90%) are very low. That explains why even the polygenic scores perform poorly (Hingorani et al., 2023). As Wald & Morris (2011) say, ‘Many risk factors for disease are suggested for screening tests when there is little prospect that they could be useful in predicting disease’. Wald & Old (2019) even go so far as to use ‘The illusion of polygenic disease risk prediction’ as the subtitle of a paper. More recently, they state that ‘to detect 80% of affected individuals at a 5% false positive rate, which would be useful in screening, requires a hazard ratio of 2284 when comparing the top and bottom quintile of biomarker distributions’ (Hingorani & Wald, 2025).

Such a large hazard ratio is practically impossible to find in clinical medicine.

Moreover, polygenic scores, created by summing together all the association scores, are only a mathematical convenience; they are, strictly speaking, fictional. In non‐linear interactive systems, the association score for each gene must depend on all the other relevant genes and the environment. Association is therefore a very poor way to assess functional significance. Genomic interpretations of association scores use complex data manipulation methods in the search for what they call ‘causation’ (e.g. Ota et al., 2026). It cannot be stated strongly enough that association alone cannot reveal causation.

Even a zero association score may hide substantial causation (Noble, 2025b). This problem has been recognized for many years. The joint discoverer of the link between smoking and cancer, Austin Hill, drew attention to it in a carefully written President's Address (Hill, 1965). The association data became convincing leads to causation only when anatomical and physiological evidence was forthcoming in the form of histopathological evidence from the bronchial epithelium and the discovery of carcinogenic factors in tobacco smoke.

In the next section, we will show how modern quantitative network physiology (Ivanov, 2021) can provide causal explanations. As Hill noted, it requires hypotheses about causation that can be tested experimentally. Without equivalent identification of causal factors in functional processes, measuring association scores cannot succeed in finding cures.

How do we achieve that? We can do so with a combination of experiments on cell and organ dynamics, combined with quantitative modelling of the processes identified. That requires an understanding of the philosophy of such modelling in biology. All theories in science imply a metaphysics inherent in the background assumptions on which the theories are based.

## 4 THE METAPHYSICS OF CAUSATION IN PHYSIOLOGY

Metaphysics is a broad field of speculation in science, because speculations are the foundations of testable hypotheses required to construct any theory of causality. Without speculation and experimental investigation, no useful theories could be elaborated. The focus of such speculation is therefore what is required to measure causation experimentally in living organisms. That then takes the form of a quantitative model of interactions between the components of regulatory networks (Noble & Bourret, 2026: fig. 1) and knowledge of the structures (membraneous and otherwise) within which those interactions occur.

There are two forms of causation here: cause by form and cause by action. A sequence that waits, like a database, to be read by other active components is causation by form, which is the molecular composition and shape. This is precisely what DNA sequences provide. As chemicals they are passive molecular threads, consisting of templates from which amino acid sequences are determined. So is the existence of membraneous structures, acting as constraints on what the molecular components can do inside and between them. In contrast, the movements and changes of shape within the elements, such as the opening and closing of gates within membrane‐bound channel proteins, is causation by action. As we showed previously (Noble & Bourret, 2026: fig. 3), any model requires simultaneous integration of these two forms of cause to obtain solutions, from which both the association and causation can be calculated and compared.

There are no useful shortcuts in such analysis, nor can the results be obtained by transformations, such as Mendelian randomization, of association scores, because genomics provides access only to static association scores. Genomic association studies use no differential equations for the molecular and other dynamic components, and no physical boundary conditions are specified. Genomics does not even attempt to quantify the complexity of biological membranes and other cellular structures. Yet, calculations on how much information is required to represent these structures show that it is at least as great as that in the genome (Noble, 2017). They form constraints that are also causal. Nor does genomics identify the dynamic processes at molecular, cellular and other levels that cause normal and abnormal function.

All that can be achieved with genomics alone, therefore, is the transformation of one kind of association score into another. In effect, there is no physical model of the causal process, hence physical causation cannot be derived.

Moreover, there can be substantial causation even when the association score is negligible or even zero. Hillenmeyer et al. (2008) showed that, in yeast, 80% of genes show zero association in favourable physiological conditions. During metabolic stress, most of the 80% that were silent then show large associations. For precisely this reason, 90% of genomic association scores are low. Yet, hidden behind those low associations lies massive latent causality. Latent, because it kicks in only when the organism needs it to do so.

The same insight arises in purely physical engineering systems. Modern aircraft are equipped with a traffic collision avoidance system that alerts the crew when a collision is possible. This system is coupled to (i.e., associated with) the autopilot of the aircraft. In flight, this system normally remains silent or passive. If two aircraft are on converging trajectories, the system will automatically make one of the two aircraft climb (or descend). The cause of this sudden change in trajectory will be the proximity of the two aircraft. There is then a dynamic aspect of causation. The traffic collision avoidance system will then actively adjust the flight controls via the autopilot, in addition to engine power as needed, and will thus act on two different functions of the aircraft according to the needs. We may speak of latent causation, because the operation of the flight control systems or the action on engine power will be activated only when needed, which is precisely how latent causation operates in living systems.

Latent causation, hiding beneath low or even zero association scores, is also important in evolutionary biology, where we need to correct an important mistake in *The Extended Phenotype*. Dawkins writes:

The *difference* between the *carbonara* and the *typica* phenotypes (of moths) can still be due to a difference at one locus, even though the phenotypes could not exist without the participation of thousands of genes. And it is the same difference that is the basis of natural selection. Both geneticists *and* natural selection are concerned with differences! However complex the genetic basis that all members of a species have in *common*, natural selection is concerned with differences. Evolutionary change is a limited set of substitutions at identifiable loci. (Dawkins, 1982: p. 93, italic emphasis in the original)

To see why this cannot be correct, consider a well‐adjusted population, in which the association score for any particular gene is zero. Would that mean that it can play no role in evolution? Clearly not, because an environmental change might occur in the future, in which the association score becomes significant, just as in the experiments on yeast. Selection can then act on a characteristic influencing the fitness of the species to survive. A zero association score in ideal conditions does not mean that it cannot be selected in future environmental change. This is also a suitable point at which to note the discovery that this difference between the phenotypes of moths was not a point mutation. It was a functional transposable element (Hof et al., 2016) rapidly induced by the stress on the phenotype (Casacuberta & González, 2013).

## 5 EXAMPLES OF PHYSIOLOGICAL CAUSATION

If genomic associations cannot be relied on, what then does identify causation? The answer is identifying the dynamic phenotype.

The Hodgkin–Huxley equations (Hodgkin & Huxley, 1952) are the classic example of a dynamic causal model in physiology. The basis of that model was the Hodgkin cycle, formed by the feedback between a cellular property, membrane potential, and the gating kinetics of the protein channels. The outcome was a correct prediction of the nerve conduction velocity and of the ionic fluxes involved. It also identified how anaesthetics work by blocking the voltage‐dependent sodium ionic channel. All of this was based on precise experimental details, on which the model equations were constructed.

The same approach was used by one of us to measure the differences between association and causation in the pacemaker rhythms of the heart (Noble et al., 1992; Noble, 2025a, 2025b). The results showed large differences between association and causation, attributable to the robustness of heart rhythm. Each process is backed up by others that kick in when the relevant gene is absent or when the protein it codes for is blocked. In turn, this led to a safe medication, ivabradine (DiFrancesco & Camm, 2004), for treating patients needing to reduce metabolic stress. From such dynamic modelling, we can also identify latent causation. What happens when one of the pacemaker channels is blocked revealed the latent causation provided by the others. Without that natural and robust safety process, it is difficult to imagine a drug like ivabradine being investigated for its therapeutic action.

None of these practical outcomes would have been possible with association scores alone from genomics. Understanding and finding solutions requires a deep knowledge of how dynamic systems work.

The metaphysics in these examples is simply which physical or chemical principles to take for granted in constructing the models. Apart from that, the data used are based on detailed experimental evidence. Furthermore, it is not possible to achieve these outcomes by claiming that ‘hypotheses are a liability’ (Yanai & Lercher, 2020). On the contrary, hypotheses are completely necessary for any causal explanation to be possible. Otherwise, we are overwhelmed with the enormity of the options amongst hundreds or thousands of data and various transformations of their association scores, none of which has involved experimental information on causation in dynamic functional networks.

## 6 THE WAY OUT OF THE GENE‐CENTRIC IMPASSE

Can we generalize these principles to outline a way out of the gene‐centric impasse that the reliance on genomics alone has created? Genomics has exhausted almost all its capacities by producing huge catalogues of data and various transformations of association scores. But it alone cannot generate physiological models that represent dynamic causality.

Constructing models in the way done in physiology, metaphysics is not mere speculation, because it leads to highly practical outcomes: the identification of active causation, which is the only secure route to finding cures. The theories developed are based on physiological experiments, ensuring that the modelling can be quantitatively accurate in its applications and predictions. It does not involve currently untestable metaphysical ideas (e.g., about the origins and nature of the universe).

To follow the examples of modelling nerve axons and heart rhythms, we need models that satisfy the following conditions:

1. The model must drill down to the level of gene products (proteins and RNAs); otherwise, there would be no possibility of mimicking a gene variation/knockout to assess an association score.
2. It must reproduce the relevant phenotype function.
3. It must conform to the basic laws of physics. CellML is constructed to test for that. The models in the CellML Repository are guaranteed to satisfy those laws of physics when formulated in bond graph form.
4. It must identify the causal factors involved in creating the phenotype by revealing what processes could be targeted to effect a cure of a disease state.

## 7 EXAMPLE OF THE COMPLETE PROCESS USING MODELS OF HEART PACEMAKER RHYTHM

Normal heart rhythm is generated by the sinus node region of the heart. This contains a few hundred thousand cells, all interconnected by nexus junctions ensuring that there is a co‐ordinated rhythm of the whole node, which then transmits excitation to the atrial cells. The expression levels of the various ionic channels vary systematically between the centre, where the maximum negative membrane potential can be as low as −50 mV, and the periphery, where the maximum negative membrane potential approaches that of the atrium, i.e., between −75 and −80 mV. Given that the i(f) channel is highly sensitive to membrane potential over the whole of this range of potentials, the ionic current contribution from this channel can vary all the way from being the main depolarizing current during the pacemaker depolarization in the periphery to making a very small contribution at the centre. When the current generated by this channel is large, the depolarization is faster. When the complete node is simulated with a multicellular network representing these characteristics, the electrical beat therefore begins at the periphery, and the wave conducts towards the centre. This was shown in computations published by Cai et al. (1994). The computations were performed on the Connection Machine, with 64 processors, each representing a single sinus node cell model.

This was a surprise result, because it is well known that in the complete heart, the excitation wave begins in the centre of the node and conducts towards the periphery. The computed result is correct, however, when the sinus node is dissected free of connection to the atrium, as shown by Toyama et al. (1995). We now use models of the central and peripheral cells to analyse the results leading to a successful drug treatment.

**FIGURE 3:**
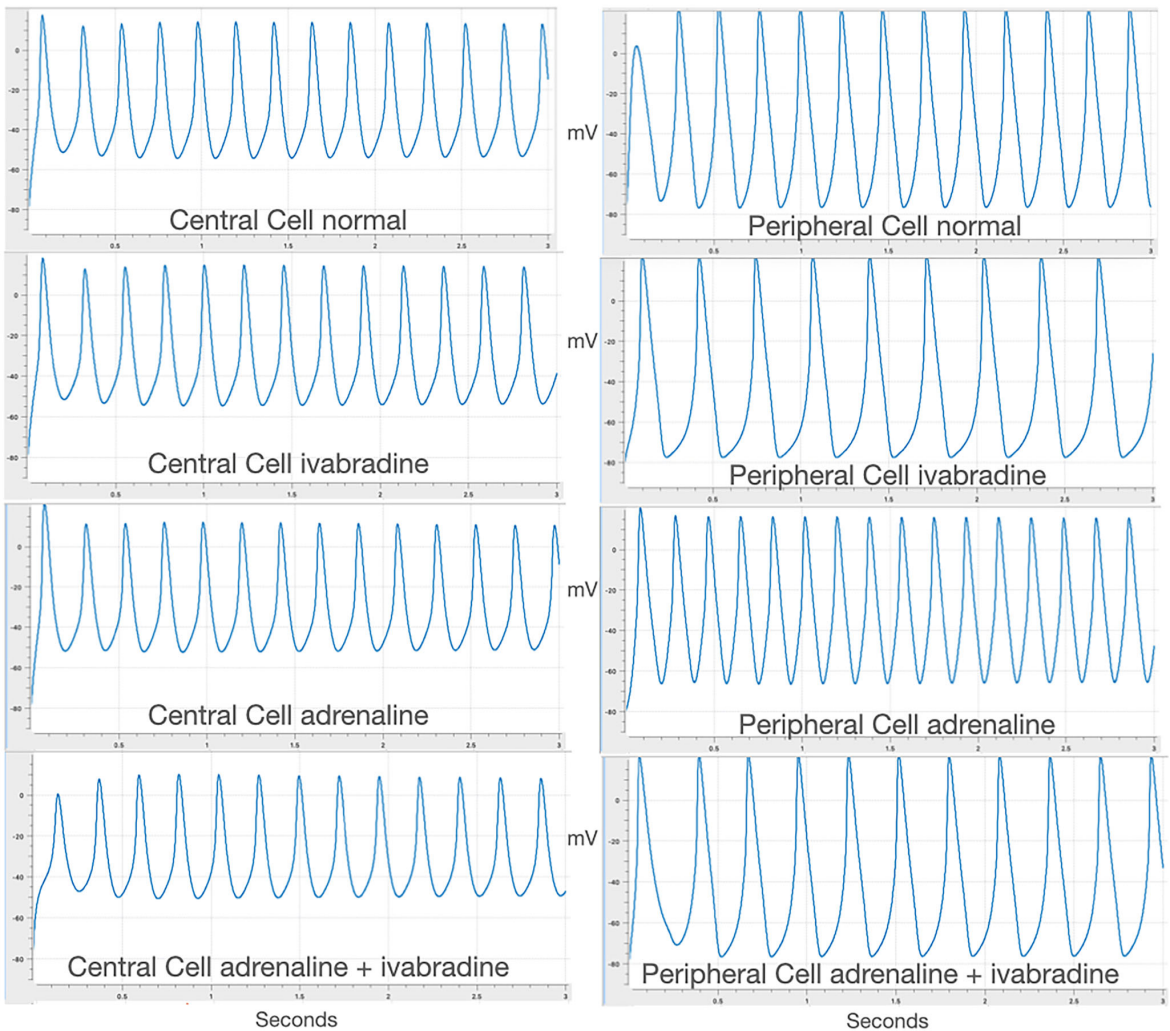
The behaviour of the Noble et al. (1992) sinus node model (top left panel) and a peripheral cell model (top right) developed by increasing the parameter gK1 to 0.15 to generate a maximum negative potential around −80 mV. The panels in the second row illustrate the action of ivabradine (procoralan) by blocking the i(f) channel by 90%. There is a much larger effect on the peripheral cell, as expected. The panels in the third row illustrate the effects of adrenaline resulting from a −15 mV shift of the i(f) activation curve. The panels in the fourth row illustrate how ivabradine would then be expected to decelerate the rhythm by a much larger effect in the presence of adrenaline. (These new computations using the 1992 model were done in May 2026 specifically for this paper.)

Figure 3 shows that from an engineering viewpoint, ivabradine is a demand controller by limiting the demand on the heart during exercise. There is no way in which associations alone could have pinpointed this neatly engineered mechanism. It is only apparent at the phenotype level.

## 8 THE GENE‐CENTRIC IMPASSE IN STUDYING THE NERVOUS SYSTEM, AND PRINCIPLE 9: THE ORIGIN OF THE SELF

In our article updating the principles of systems biology (Noble & Bourret, 2026), we omitted the last two principles, 9 and 10, in the paper by Noble (2008). We did so because principles 1–8 concern interactions between multiple levels of physical organization in organisms and can be given purely physical interpretations, whereas principle 9, which states that ‘The Self is not an Object’, clearly implies non‐material interactions. We delayed revision of that principle because it is easier to do so after first identifying the gene‐centric impasse, then showing how physiology can lead the ways out of the impasse. As we will now show in this section, association studies on nervous diseases serve to highlight the great importance of principle 9. The self is not an object entirely within the organism. It is a process that necessarily includes social interactions with other organisms. Yet the idea that the conscious self is an object within the nervous system (Crick, 1994) led to nervous diseases forming a key element in the confidence that the Human Genome Project would lead to cures. Schizophrenia and Alzheimer's disease were selected as prime illustrations. James Watson (1990) wrote: ‘the messages encoded by our DNA will not only help us understand how we function as healthy human beings, but will also explain, at the chemical level, the role of genetic factors in a multitude of diseases, such as cancer, Alzheimer's disease, and schizophrenia, that diminish the individual lives of so many millions of people’.

Schizophrenia is well known to run in families and was for that reason confidently expected to yield to genetic explanations.

But, commenting on progress up to 2024, Torrey (2024) wrote:

One of the most serious diseases that was specifically targeted by the Human Genome Project was schizophrenia. A recent survey reported that it affects approximately 4 million American adults based on 2020 census data. The annual economic burden of schizophrenia in the US, assuming 3.9 million individuals affected and using 2019 data, is $97.3 billion in direct costs and another $251.9 billion in indirect costs, making it one of our most expensive diseases. Alzheimer is predicted to cost around $781 billion for 2025, and projections nearing $1 trillion by 2050.

These costs are unsupportable. That is true in all developed nations around the world.

Torrey concluded:

In summary, the human genome project was undertaken to discover the genetic causes, and ultimately better treatments for many diseases, prominently including schizophrenia and other serious psychiatric disorders. Since the initial results became available in 2000, NIMH [National Institute of Mental Health] has spent almost $8 billion in pursuit of this goal. Not a single gene has been found that can be causally linked to schizophrenia and the research has produced no improvements in treatments. Thus there is no evidence that faulty genes cause schizophrenia. Like almost all human diseases, risk genes have been found and play a role in the clinical expression of schizophrenia but they do not cause the disease.

If not genetics, then what does explain the fact that the illness runs in families? The answer is social deprivation, which also often runs in families. In their extensive study of investigations on the social determinants of health and disease, Jester et al. (2023) reported:

Childhood abuse, parental psychopathology, parental communication problems, bullying, and urban settings with lower socioeconomic status were major risk factors for the greater incidence of SSPD [schizophrenia‐spectrum psychotic diseases] and/or worse health. Social network size was inversely associated with overall psychopathology and negative symptoms. Experiences of racial/ethnic discrimination correlated with the prevalence of psychotic symptoms and experiences. Compared to native populations, the risk of psychosis was higher in immigrants, refugees, and asylum seekers. Social fragmentation was associated with an increased prevalence of schizophrenia. Homeless populations had a 30‐fold higher prevalence of schizophrenia than the general population.

Diseases such as schizophrenia and Alzheimer's disease are better viewed as diseases of the mind. In the severest forms, they seriously affect the sense of self and, by so doing they degrade its sense of worth and well‐being, thus incapacitating the individual involved.

## 9 PRINCIPLE 10: THERE ARE MANY MORE TO BE DISCOVERED

Of course, this is not a principle of systems biology, it is a principle applicable to all science. We must be open to what our experiments tell us. At various levels and in different fields of research, recent results demonstrate the need to move beyond what was thought to be the only path. For instance:

1. At the level of the origin of life, by examining which types of polymers are the most suitable while also being the simplest. RNA polymerase ribozymes can catalyse and copy. This activity is possible with only 45 nucleotides. However, the first active sequences were logically short, hence the interest in demonstrating their activity, as Gianni et al. (2026) did by working with QT45, which can copy a variety of different RNA templates, including sequences with tightly folded secondary structures and those encoding a hammerhead endonuclease ribozyme.
2. At the level of dynamics of kinetics, the continuous instantaneous kinetics must be analysed and would be a normal continuation of the work on longitudinal transcriptomics, which is a point‐in‐time technique (Yadav et al., 2018) that shows moderate correlations between mRNA and protein profiles (Yang et al., 2025). Proteome analysis, more important for predicting gene function (Wang et al., 2017), and methods for long‐read sequencing having a higher base‐calling error (Xu et al., 2025) should not be excluded, nor should any methods that can define causal factors in complex systems, provided that the data are not highly manipulated by metadata processing ((Serazetdinova et al., 2025).
3. At the level of complex molecule synthesis, starting from DRT3 (defense‐associated reverse transcriptase), whose DRT3b component can synthesize itself and its complementary strand (Deng et al., 2026). The protein itself serves as the blueprint for the DNA. The title, ‘protein‐templated synthesis of dinucleotide repeat DNA’, is already challenging. They have, in fact, received much criticism and many questions about their results. This is one of the avenues to explore.
4. At the level of embryonic development, the early genome organization is orchestrated by an interplay of overlapping yet separable regulatory inputs, which appears before zygotic genome activation, and both factor‐specific mechanisms and broader context‐dependent constraints are involved in proper genome establishment (Maziak et al., 2026).
5. At the level of populations (and this could also be searched for other species): the directionality of selection (i.e., purposefulness) has affected several hundred alleles. And the ‘frequencies of variants shifted more than could be expected by chance’. This was shown in a study of >15 000 individuals over the last 10 000 years (Akbari et al., 2026).

**FIGURE 4:**
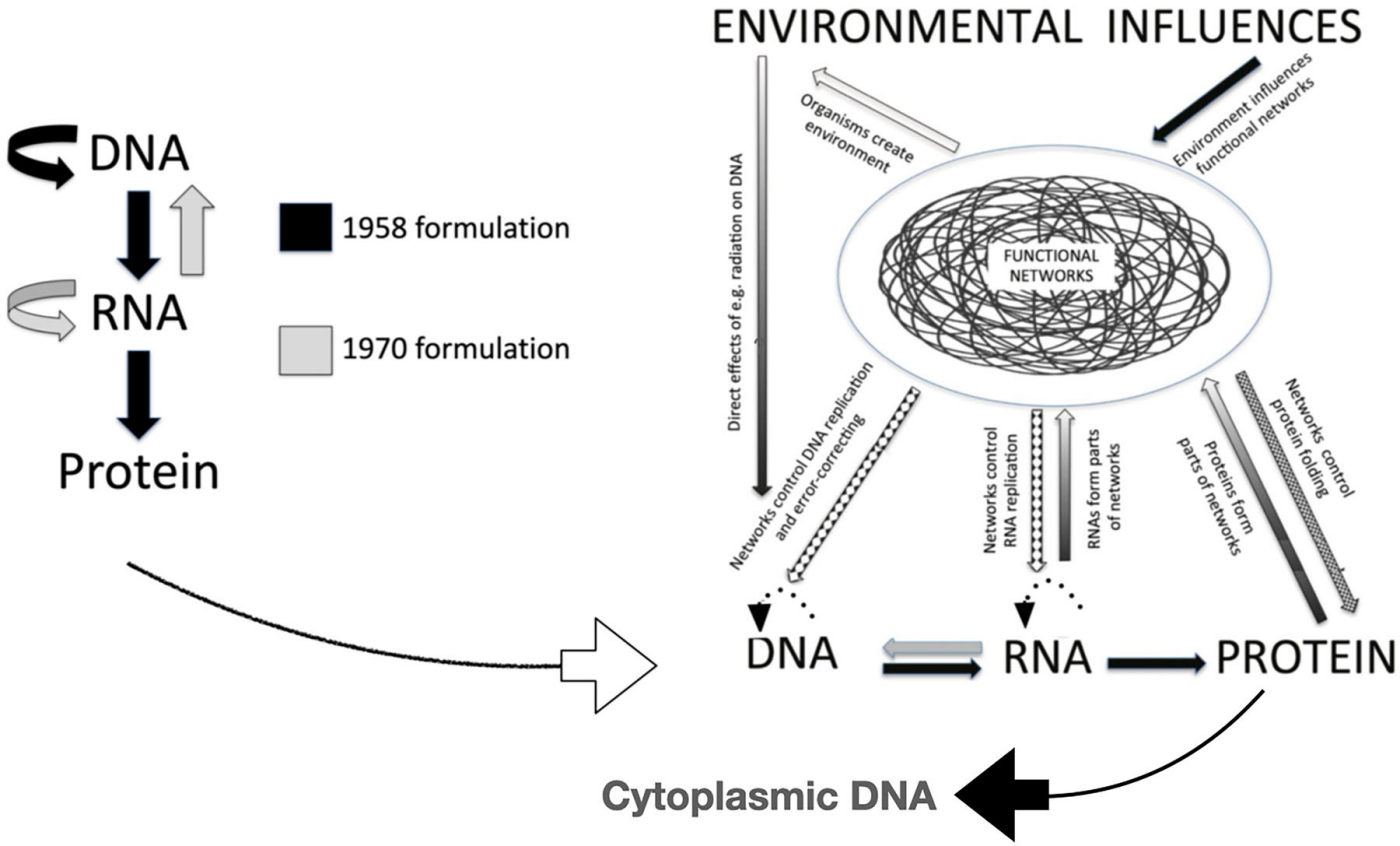
Figure 1 from Noble & Bourret (2026), with the addition of an arrow from PROTEIN to cytoplasmic DNA.

The results of Deng et al. (2026) challenge a part of the central dogma that looked secure. We have therefore added an arrow from protein to cytoplasmic DNA to figure 1 from Noble & Bourret (2026) to represent what their results show, to form Figure 4. The additional arrow points to cytoplasmic rather than inherited DNA, because their results show the formation of a functional DNA enabling bacteria to neutralize phages, not necessarily to edit inherited DNA.

In recalling the history of science, the history of the idea of the ‘genetic code’ is a prime example of principle 10. Many more may be discovered because there should be no dogmas in science. As we will now show, that is also where yet another fundamental error developed in formulating the central dogma, i.e., the confusion between syntactics and semantics.

## 10 THE SYNTACTICS OF DNA SEQUENCES DO NOT REVEAL SEMANTICS

Over and above the difference between association and causation, there is that between syntactics (grammar) and semantics (meaning). Unfortunately, the language adopted to describe the nucleotide sequences confused these two completely different interpretations of those sequences. As their impressive molecular biological work developed from the discovery of the double helix into formulating an information theory account of DNA, Watson and Crick failed to distinguish between metaphorical and literal meanings of the word ‘code’. This is what led to the idea that the chemical sequences in DNA could be regarded as the ‘book of life’, the ‘code’ for creating a living organism. They either forgot or deliberately ignored the difference between syntactics and semantics. A possible reason is that they assumed, following Schrödinger (1943), that DNA is a perfectly accurate self‐replicator. There would then be no way in which the living cell could be imagined to change genomic sequences. As we have explained (Noble & Bourret, 2026: fig. 1), that cannot be true because DNA is very far from being an accurate self‐replicator. We also showed that when the genome is paired up with an egg cell of another species  to form a cross‐species clone, it fails to determine the correct anatomy. The number of vertebrae fell between that for the two species, carp and goldfish.

Without the help of a living cell and its coordination of a set of cut‐and‐paste enzymes, the G2 step, allowing a cell to divide by passing highly accurate DNA copies to the daughter cells, would never be achieved. This mistake also led to Jacob & Monod (1961) comparing DNA to the program containing the computer code for creating the living organism. That analogy was explicitly stated as equivalent to inserting paper or magnetic tape into the mainframe computers of those days. François Jacob (1982) wrote: ‘The programme is a model derived from electronic computers. It equates the genetic material with the magnetic tape of a computer’. The work of one of us (D.N.) in the 1960s and 1970s used such tapes to enable the computations that led to the first cardiac cell electrical models (Noble, 1960, 1962). The analogy is deeply misleading, because the complete living cell is necessary for the analogy to work. This is equivalent to expecting the computer itself to generate and run the code to boot it up. DNA alone cannot be used to create a living organism. It needs an egg cell to do so. The computer needs its operating system.

DNA is a passive chemical, not an active one. Its sequences need to be read by a living cell to form the chemically active molecules, RNAs and proteins. The reading of the DNA sequence to create RNAs is the process of transcription.

The history of the development of the language used to describe DNA and its transcription has been researched extensively by the historian and scientist Lily Kay (1993, 2000). Geneticists initially recognized the metaphorical use of ‘code’ and ‘program’ by including the quotation marks. It was Watson & Crick (1953) who first dropped those critically important quotation marks. When Crick went on to formulate his central dogma in 1958, he not only dropped them, but he also presented the arrow from RNA to protein as irreversible, thus implying that there could be no way in which living organisms could change their DNA. He did so by implication, because his diagrams never included downward causation from the functional networks. By also assuming, following Schrödinger (1943), that the genetic material replicates faithfully, ‘like a crystal’, his central dogma became widely understood to forbid such change. Crick and many other DNA researchers then thought that they were naturally applying the language of information theory (Watson & Crick, 1953) to biology. Yet information theory itself deals only with syntactics. Whether the code it processes, to encrypt or decrypt any message, has any meaning is strictly irrelevant to data communications: As Kay notes ‘It always makes cryptographers indignant to hear the terms “code” and “cipher” used synonymously’. Kay (2000: pp. 151–152).

There was, however, still hope that this deep error could have been corrected. Jacques Monod was ‘not a naïve participant in the information discourse. Fully aware of the general features of the mathematical theory of communication—its scope and limits—he knew within that domain information was purely syntactic and devoid of semantic value’ (Kay, 2000: p. 220). He also wrote to Brenner in April 1959: ‘nuclear replication as well as protein synthesis are strictly controlled by the cell’ (Kay, 2000: p. 224).

This unnecessary restriction of ‘information’ to nucleotides is also irrelevant to the chemistry and physics of living organisms. Those nucleotide sequences are interpreted by living organisms in the context of all their significant processes. Sequences that once ‘coded’ for flippers and fins in sea creatures eventually became used to ‘code’ for terrestrial limbs. Even ‘coding’ for a protein such as aconitase does not ensure the same function. That depends on whether the protein finds itself in free solution, acting as an enzyme, or in a cell membrane, acting as an ion channel (Ball, 2023: pp. 151–152). It is life itself that interprets the information it receives in all its many forms. The rest of inheritance from the egg cell is necessary to the development of the embryo.

The correct term for the relationship between one form of text (nucleotide sequences) and another (amino acid sequences) is ‘intertextuality’ (Kay, 2000: p. 283). DNA is not a code for decrypting the sequences of proteins, or vice versa. To use the word ‘code’ for this intertextual relationship is to try to invent a ‘code for codes’. There is no definition of what this expression could even mean. Furthermore, there is no reason to single out DNA as the sole carrier of inherited information:

all organized entities—carbohydrates, proteins, nucleic acids [to which she could have added membranes]—contained information. Molecular geneticists … singled out nucleic acids as the unique carriers of informational attributes. Information—as meaning and commodity—came to signify the privileged status of DNA as “master molecule.” Emptied of its technical content, it actually became a metaphor of a metaphor, a signification without a referent. (Kay, 2000: p. 127)

Does all of this careful analysis of the language of biology matter? Yes! Misuse of the language of information theory by gene‐centric biologists led inevitably to the gene‐centric impasse, by promising more than could ever be delivered. ‘The genomic visions are simplistic, promising a great deal more than can reasonably be delivered’ (Kay, 2000: p. 326).

## 11 DISCUSSION

There seems to be no way through the gene‐centric impasse in health care through further analyses of genomic association scores. The reason is that association studies do not measure the relevant dynamic and static causes of biological functions. The dynamic causes include physiological interactions at all levels of organization. The static causes include the anatomical and organizational constraints under which those dynamic causes operate. Association studies also do not detect latent causes. Even zero association scores can then hide major latent causation.

It is no exaggeration to say that the misuse of the language of information theory by molecular biology, so precisely pinpointed by Kay (2000), together with the ignoring by genomics of the mathematical logic of Wald and his colleagues on the criteria for successful screening, has delayed for ≥30 years understanding the inevitable conclusion, which is biology must return to studying the physiology of the phenotype to avoid the expensive mistakes leading to the gene‐centric impasse. The warnings from the works of Kay and Wald were already clear in the mid‐1990s, three decades ago.

This is a clarion call to the biological community in general. It is urgent to wake up to the failures of the clearly stated, but equally clearly unattainable, objectives of the Human Genome Project. We will have to educate a whole new generation of biologists to understand how dynamic physiological processes are measured and how they can lead to clinical discoveries of importance in the treatment of multifactorial diseases dominating poor health in the ageing population.

We have outlined the way forwards, which is to measure dynamic and static causes accurately enough to construct dynamic experimentally based models. These can then be used to explore interventions for possible therapeutic effect. Our new numerical analysis of the actions of the medication ivabradine illustrates how useful cures for fatal diseases may then be identified.

## AUTHOR CONTRIBUTIONS

Both authors contributed to all stages in the writing of this article.

## CONFLICT OF INTEREST

None declared.

## FUNDING INFORMATION

None.

## GENERATIVE AI STATEMENT

 There was no use of Generative AI.
